# Global food trade alleviates transgressions of planetary boundaries at the national scale

**DOI:** 10.1016/j.isci.2023.107794

**Published:** 2023-08-30

**Authors:** Xiawei Liao, Ao Liu, Li Chai

**Affiliations:** 1 Bay Area International Business School, Beijing Normal University, Zhuhai 519087, China; 2 College of Economics and Management, China Agricultural University, Beijing 100083, China; 3 International College Beijing, China Agricultural University, Beijing 100083, China; 4 National Innovation Center for Digital Fishery, China Agricultural University, Beijing 100083, China

**Keywords:** Agricultural land, Environmental science, Food policy, Sustainability aspects of food production

## Abstract

Food systems are among the leading causes for transgression of planetary boundaries globally, which define the safe operating space for humanity. We quantify unsustainable environmental impacts of food systems, indicated by the transgression of national-scale planetary boundaries (i.e., the safe operating space for food production in each country), from both production and consumption perspectives of 189 countries/regions around the world. A multi-regional input-output model is used to map the global transfers of the national-scale transgression of planetary boundaries, including freshwater use, land change, and biogeochemical flows (nitrogen and phosphorus). Our results show that China is a major global unsustainable water and nitrogen exporter and an unstable land and phosphorus importer. This means that water and nitrogen uses in China are used to support food demands in other countries, and food consumption in China requires unsustainable land and phosphorus uses elsewhere. In contrast, the US is a major exporter of unsustainable water, land, and nitrogen uses but only an importer of unsustainable phosphorus for food consumption. Globally, compared to a counterfactual scenario where there is no food trade among any countries, food trade saves massive transgressions of planetary boundaries (270 km^3^ of water, 18 million tons of nitrogen, 7 million tons of phosphorus, and 5,431 million km^2^ of land). Alleviation of national-scale planetary boundary transgression has been achieved primarily in the US, China, Saudi Arabia, etc., while aggravation was incurred in Pakistan, Australia, Argentina, and so forth.

## Highlights

• Unsustainable water and nitrogen use in China and the US support food demands elsewhere
• Globally, food trade avoids substantial national planetary boundary transgressions
• Trade alleviates national planetary boundary transgressions in the US and China
• Trade aggravates national planetary boundary transgressions in Pakistan and Australia

## Graphical abstract

**Figure:**
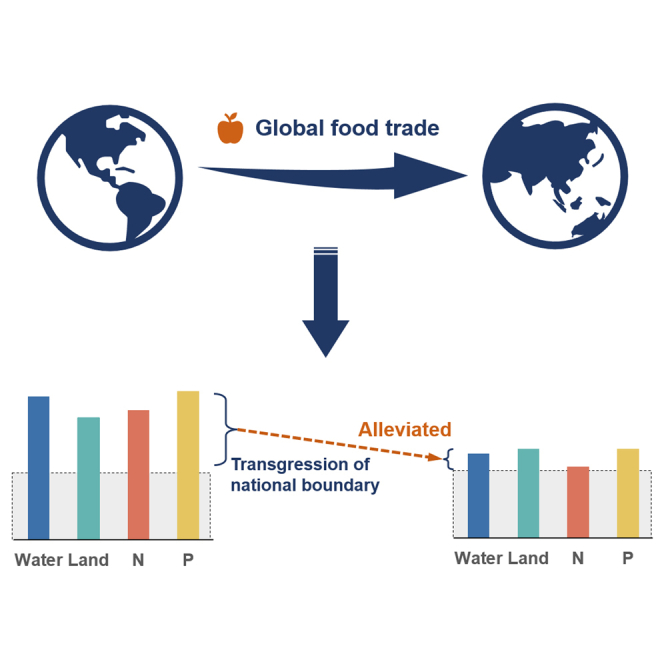

## Introduction

The concept and framework of planetary boundaries propose sustainable limits of nine human-biophysical systems or processes as safe operating space within which humanity can continue to develop and thrive.^1^ Those nine processes include climate change, biosphere integrity, global biogeochemical flows (nitrogen and phosphorus), stratospheric ozone depletion, ocean acidification, global freshwater use, land use change, chemical pollution, and atmospheric aerosol loading. Substantial and persistent transgression of those boundaries driven by anthropogenic activities is expected to have detrimental, if not catastrophic, consequences on our world. According to most up-to-date assessment, six out of the nine boundaries have been transgressed, including climate change, biosphere integrity, biogeochemical flows, land system change^2^ and, most recently in 2022, novel entities, including plastics, and freshwater use.^3^^,^^4^

It should be noted that those nine systems operate at different scales, from local catchments to the entire earth system. Rockstrom et al. (2009)^1^ recognized that while some earth systems, such as stratospheric ozone depletion, ocean acidification, and climate change, should be studied at a global scale,^5^ control variables of several other systems are spatially heterogeneous, most notably freshwater use, land use change, biogeochemical flows, and biosphere integrity, which therefore warrant assessments at local to regional scales. For instance, by breaking down the world into 0.5° grid cells, Gerten et al. (2013)^6^ found that although global freshwater use was still within the planetary boundary back then, many places had already shown transgression of their local planetary boundary for water.

Production of food to meet humankind’s rising food demand is a major contributor to the transgression of those local boundaries since agricultural activities in many regions are using too much water, land, or fertilizer.^7^ Occupying 40 percent of the global land surface, agricultural production is the most land-intensive production form.^8^ It is recognized as the driver for over 80 percent of the deforestation worldwide from 2000 to 2010.^9^ Besides being the world’s largest water user, accounting for over 70 percent of the global freshwater use, modern agriculture is also a major cause of human modifications of the nitrogen cycles and phosphorus flows due to the unsustainable use of fertilizer. Campbell et al. (2017)^10^ assessed that agricultural activities already account for 80 to 90 percent of the planetary boundaries for land, water, nitrogen, and phosphorus flows. Springmann et al. (2018)^11^ assessed the available boundaries for the global food systems, including 12.6 million km^2^ of cropland use, 69 teragrams (Tg) of nitrogen application, and 16 Tg of phosphorus application.

Meanwhile global trade of agricultural and food products may change the pattern of those local problems. For example, by importing water-intensive wheat-based and corn-based products from water-abundant countries, such as Brazil, water-scarce countries in the Middle East seek to alleviate their local water stress.^12^^,^^13^ Resource inputs, e.g., water and land, and nutrition modifications, e.g., nitrogen and phosphorus, can be embodied in the traded products and transferred across borders as virtual flows. Numerous studies have tried to map the global flows of virtual water,^14^ nitrogen,^15^ phosphorus,^16^ and land^17^ embodied in agricultural trade.^18^

However, few studies have examined whether those embodied environmental impacts are sustainably within the planet’s carrying capacity especially at the place of production, indicated by their local or national planetary boundaries.^19^ Gerten et al. (2020)^20^ for the first time downscaled the planetary boundaries to regional level and examined regional transgressions from food production as well as how they could be avoided. More recently, studies have linked consumption-based environmental footprint studies with research on planetary boundaries^21^ attempting to quantify how human consumption in one place incurs transgression of local planetary boundaries elsewhere.^22^

This study thus makes novel contribution by exploring the global transfers of transgression of planetary boundaries downscaled to the national level, i.e., national boundaries, to meet growing human food demand combining the downscaling of global planetary boundaries and quantification of consumption-based environmental footprints. How the global transfers of national boundary transgressions have helped alleviate or aggravated those national boundary transgressions have also been examined. Although Vanham et al. (2019)^5^ have pointed out that freshwater use, land use change, biogeochemical flows, and biosphere integrity should be studied at local to regional scales due to their spatial heterogeneity, same as Springmann et al. (2018),^11^ planetary boundaries for land use change, freshwater use, and biochemical flows are selected for this study due to the difficulties in assessing biosphere integrity indicators as well as in estimating the agricultural impacts (Campbell et al. 2017).^10^ Furthermore, with scientific advancement, biosphere integrity is increasingly recognized as a global issue, same as global climate change mitigation, that needs being evaluated at a global scale and global concerted efforts for addressing.^23^

## Results

### Transgression of national boundaries for food production

**Figure 1:**
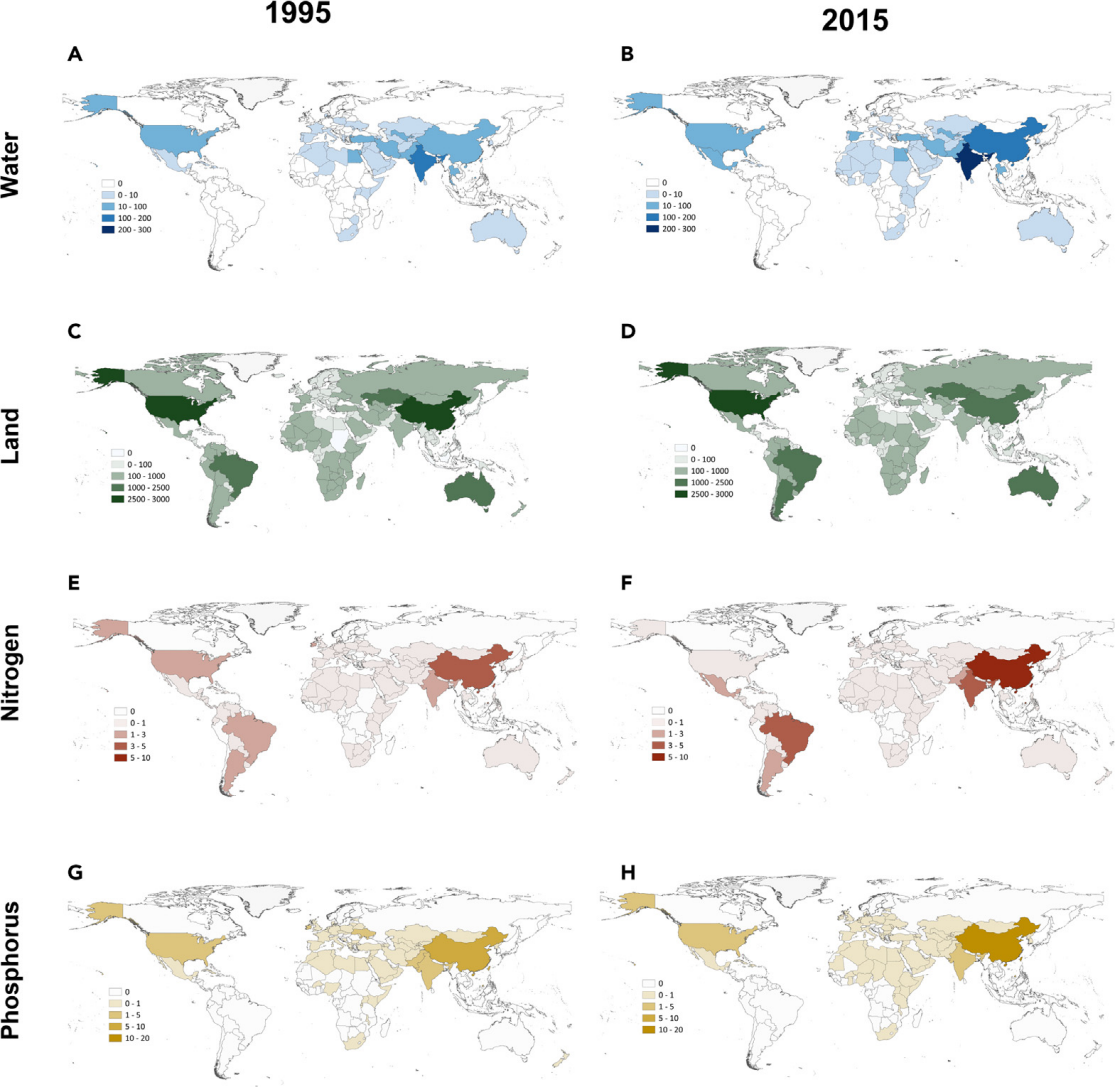
Unsustainable environmental impacts transgressing national boundaries for food production Note: Unsustainable water use in 1995 (A) and 2015 (B); Unsustainable land use in 1995 (C) and 2015 (D); Unsustainable nitrogen application in 1995 (E) and 2015 (F); Unsustainable phosphorus application in 1995 (G) and 2015 (H).

Global unsustainable freshwater use for food production has increased substantially from 568 km^3^ in 1995 to 713 km^3^ in 2015, with top 10 countries occupying almost 90 percent. India has used 276 km^3^ of water beyond its national boundary for food production in 2020, primarily for cultivation of wheat and paddy rice, accounting for almost 39 percent of the global total, followed by China (111 km^3^), Pakistan (74 km^3^), the US (50 km^3^), and Egypt (45 km^3^), which remained the top 5 countries with the largest amounts of unsustainable water use for food production in both 1995 and 2015 (Figure 1).

Similarly, global unsustainable nitrogen and phosphorus applications for food production have also increased from 21 and 22 million tons, respectively, in 1995 to 31 and 27 million tons in 2015, by a larger margin for nitrogen. China is the world’s largest unsustainable nitrogen user from food productions, followed by India and the US, making up an increasing global share from nearly 30 percent (6 million tons) in 1995 to almost 50 percent (15 million tons) in 2015, primarily to grow vegetables, paddy rice, grains, and wheat. China (6 million tons), India (4.6 million tons), and Brazil (4.5 million tons) were the three largest unsustainable phosphorus users for food production, together making up 55 percent of the global total. Besides vegetable and paddy rice cultivation in India and China, cattle farming is also a main contributor of unsustainable phosphorus application in Brazil.

The US, China, Brazil, Australia, and Kazakhstan were top five countries where food production has occupied the largest amounts of unsustainable land use in both 1995 and 2015, together accounting for more than 30 percent of the global total, primarily used for cattle farming. Another two issues worth noting are the following: first, top ten countries contributed about 57 percent of the global total; secondly, unlike the other three planetary boundary indicators, global total of national unsustainable land use for food production has decreased from 28 million km^2^ to 23 million km^2^.

### Consumption-based transgression of national boundaries

**Figure 2:**
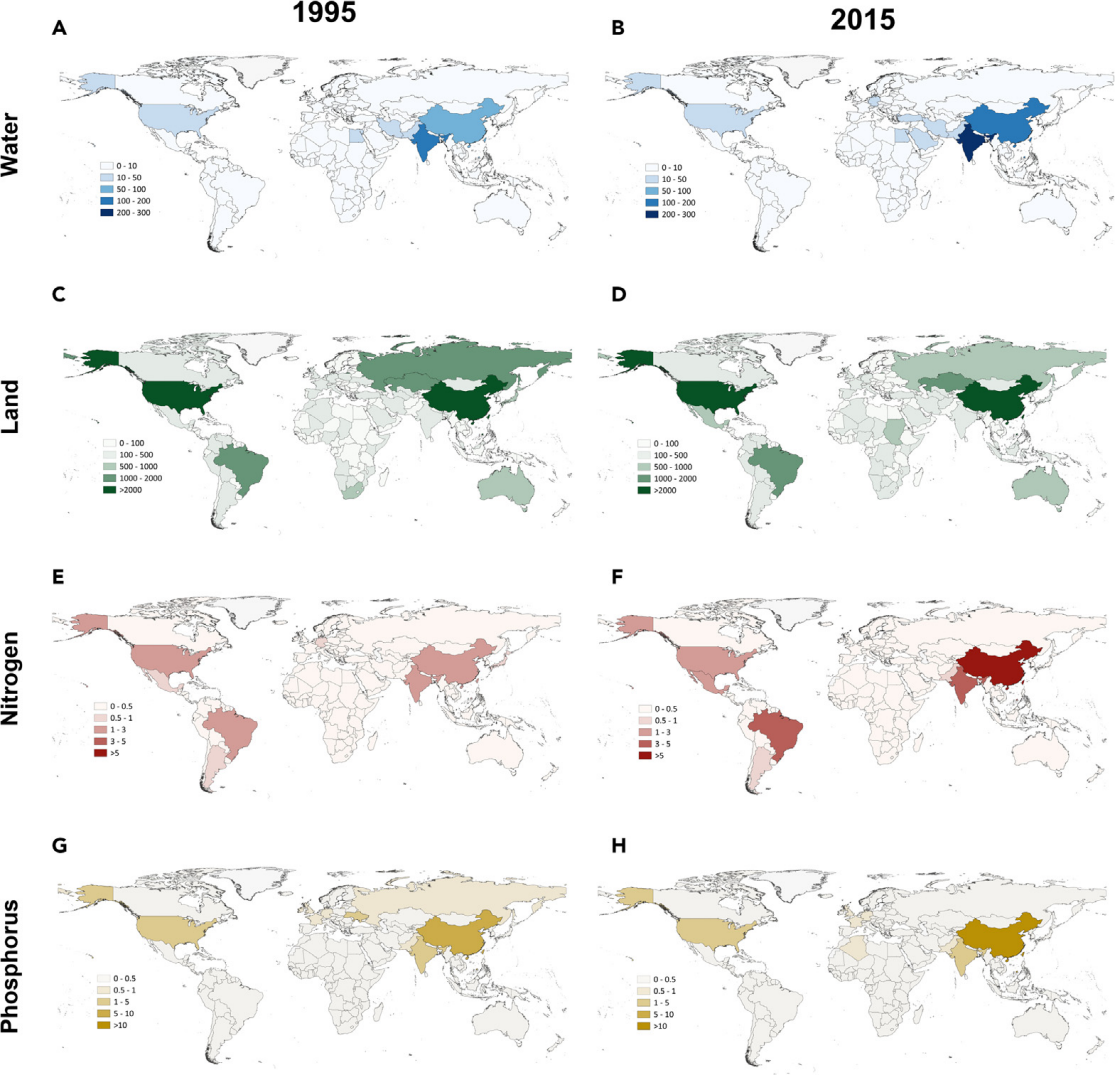
Consumption-based unsustainable environmental footprints transgressing national boundaries Note: Unsustainable water footprint in 1995 (A) and 2015 (B); Unsustainable land footprint in 1995 (C) and 2015 (D); Unsustainable nitrogen footprint in 1995 (E) and 2015 (F); Unsustainable phosphorus footprint in 1995 (G) and 2015 (H).

Increases in food production and its unsustainable environmental footprints, defined as environmental impacts along the life cycle supply chain, are incurred to support food consumption both domestically and from other countries. We quantify the extent to which food demands in one country are produced from supply chain that require excessive and unsustainable use of national resources (land and water) and nutrition flows (nitrogen and phosphorus) either domestically or elsewhere in other countries. As shown in Figure 2, our results show that India and China required the largest amounts of unsustainable water footprints, i.e., 257 and 117 km^3^, respectively, around the world to meet their respective food demands, followed by the US (49 km^3^) and Pakistan (42 km^3^). Food demand of the Chinese population is produced with the largest unsustainable nitrogen footprints globally (14 million tons), which made up 44 percent of the global total, followed by India and the US.

Different from water and nitrogen, where China, India, and the US led the rank, Brazil accounted for more food-driven unsustainable phosphorus footprints than the US. Similarly, food demands in China, the US, and Brazil required, respectively, 4, 3, and 1 million km^2^ of land transgressing national boundaries around the world, largest among all countries studied.

However, as food security is considered as a basic human right, consumption-based national boundary transgression should also be viewed in per capita terms. For example, every Chinese person’s food consumption required 80 m^3^ of unsustainable water footprints transgressing national boundaries around the world, equaling to the world average, while equaling to 40 percent of that of Indians (196 m^3^) and half of that of Americans (153 m^3^). In contrast, China has the highest per capita unsustainable nitrogen footprints (10 g per capita), more than twice of the global average (4 g per person). From 1995 to 2015, consumption-based unsustainable footprints per capita have increased for all indicators around the world, with country rankings remaining stable.

### Global transfers of national boundary transgressions for food

**Figure 3:**
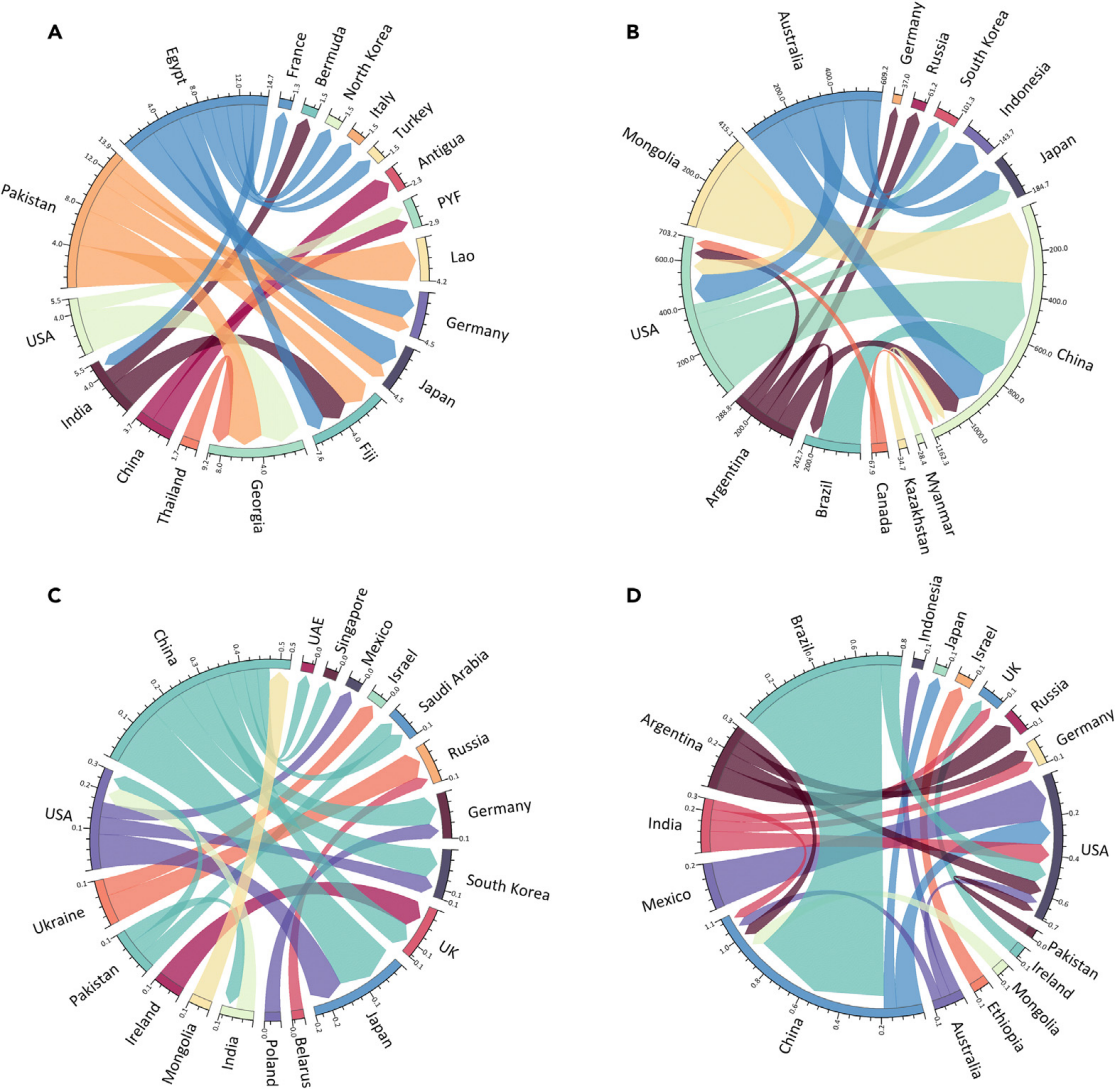
Food-driven global flows of transgression of national boundaries for: (A) water; (B) land; (C) Nitrogen; (D) Phosphorus in 2015 Note that, for clarity, we display only the top 20 links.

As shown in Figure 3, food exports had resulted in the largest amount of National Boundary for Water (NBW) transgression in Egypt and Pakistan in 2015, followed by the US, China, and India. On the other hand, food consumption in Georgia had resulted in the largest amount of NBW transgression elsewhere, followed by Fiji and Germany, demonstrating that some import-dependent countries may have small production-based transgressions, but with significant transboundary impacts on other countries. The largest three flows of NBW transgression were from Pakistan to Laos (4 km^3^), the US to Georgia (4 km^3^), and Pakistan to Georgia (3.5 km^3^).

It is noteworthy that compared to 1995, the major unsustainable water use exporters remained almost the same, including China, Egypt, India, Pakistan, Thailand, and the US, who are major water-intensive food exporters, exporting rice, soy, fruits, and so forth. However, the composition of importers has substantially diversified. In 1995, the major importers were almost all large and developed economies, including the US, Japan, Saudi Arabia, Germany, Italy, the UK, Russia, etc, while in 2015, a number of emerging and less developed economies have emerged as primary contributors to transgression of NBW elsewhere.

Australia, Mongolia, the US, Argentina, and Canada have remained the five major exporters of unsustainable land use for food productions in both 1995 and 2015. The major importers also remained almost the same, including China, the US, and Japan. Japan and the US were the primary importers in 1995, while China solely dominated the imports in 2015. In 2015, top four unsustainable land flows were all exported to China, from Mongolia (341 km^2^), the US (292 km^2^), Brazil (188 km^2^), and Australia (163 km^2^). In total, China accounted for 8 out of the top 20 flows, as well as about 57 percent of the transferred amounts.

Although China dominated the production-based unsustainable nitrogen uses (section 3.1), large amounts of those were used to produce food to support consumption in other countries, for instance, Japan, South Korea, and Germany. Similarly, transgression of the National Boundary for Nitrogen (NBN) in the US is also incurred to support food consumption in Japan, South Korea, and Mexico. In contrast, food demands in China and the US are produced from production activities that require unsustainable phosphorus applications in Brazil, India, Argentina, and so on.

### Alleviation or aggravation of unsustainable impacts of the food systems

**Figure 4:**
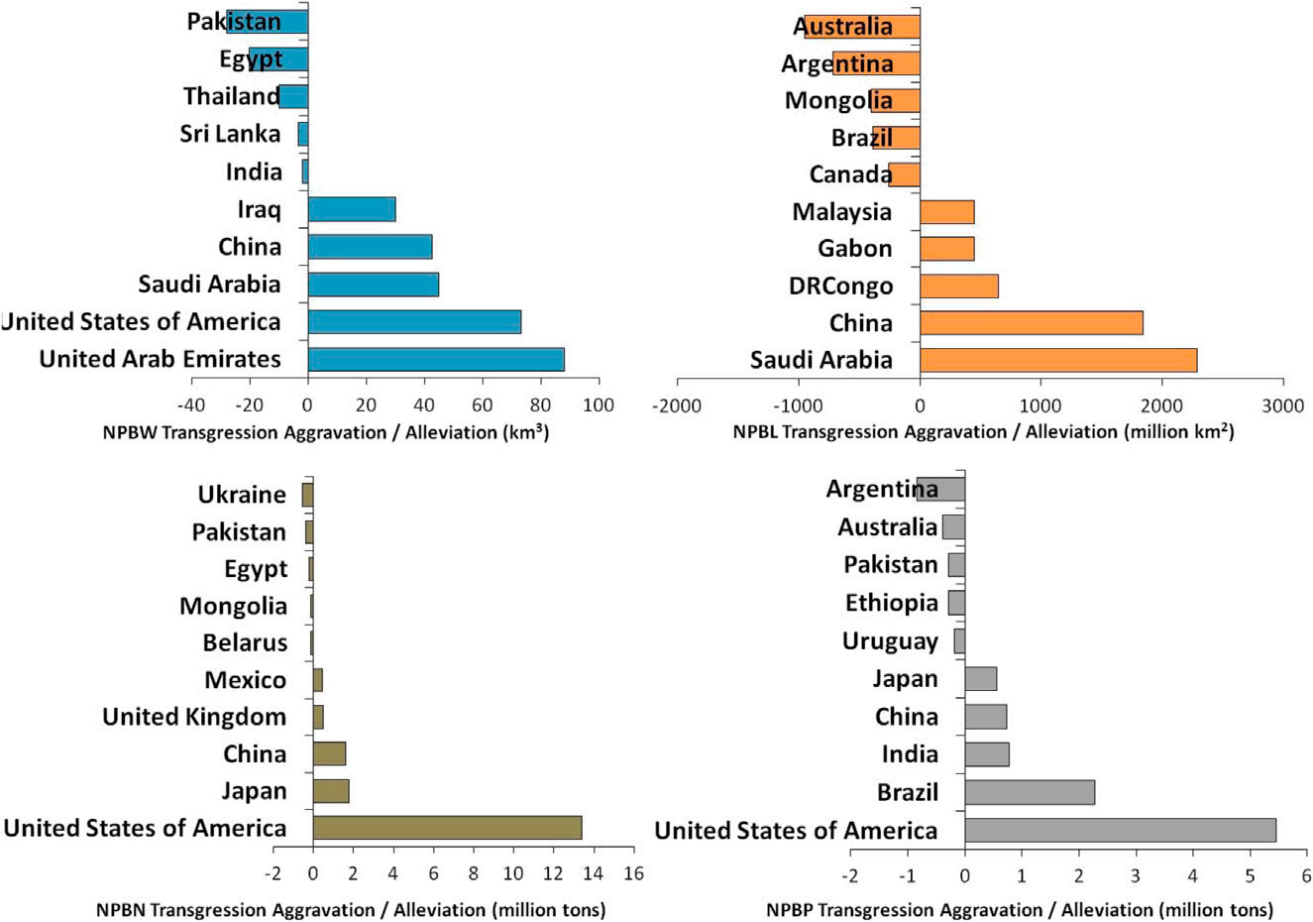
Aggravation or alleviation of national boundary transgression due to global food trade

We compare the transgression of national boundaries for food systems with a counterfactual scenario where no global food trade system exists and all countries are self-sufficient in food. As shown in Figure 4, the US, among all countries, benefitted the most from global food trade, alleviating its transgression of NBW by 73 km^3^, NBN by 13 million tons, and National Boundary for Phosphorus (NBP) by 5 million tons. Next to the US, China also benefitted from the global food trade system reducing its NBW transgression by 43 km^3^, NBN transgression by 2 million tons, NBP by 1 million tons, NBL by 1,839 million km^2^. Saudi Arabia reduced 2,287 million km^2^ of NBL transgression, the highest among all countries through global food trade. The UAE had the largest NBW transgression alleviation, amounting to 88 km^3^ in 2015.

On the other hand, national boundary transgressions in some countries, including Pakistan, are to support the global food trade system. An NBW transgression of 28 km^3^ in Pakistan is used to produce food products that are exported to other countries. Such model findings are in line with several previous studies. For instance, Dalin et al. (2017)^24^ pointed out that, as the largest net exporter of virtual groundwater, Pakistan’s groundwater security is highly threatened. Ali et al. (2019)^25^ found that the blue water embodied in Pakistan’s rice export increased from 3 to 10 Gm^3^ from 1990 to 2015. Global food trade has resulted in 949 million km^2^ of additional NBL transgression in Australia, the largest among all countries, followed by 713 million km^2^ in Argentina.

However, on a global level, food trade has avoided 270 km^3^ of unsustainable water use worldwide, 18 million tons of unsustainable nitrogen use, 7 million tons of unsustainable phosphorus use, and 5,431 million km^2^ of unsustainable land use for food compared to a counterfactual scenario where all foods consumed are produced domestically (Table S3).

## Discussion

### Consistency with existing studies

Our results are largely aligned with previous studies on global trade of virtual water and virtual scarce water, showing that India, China, and the US used the largest amounts of unsustainable water transgressing their NBW for food production^14^^,^^26^ as well as future trajectories modeled by Graham et al. (2020).^27^ Meanwhile China, the US, and Brazil used the largest amounts of unsustainable land. This is also in line with quantifications by Qiang et al. (2020)^17^ on global trends in virtual land trade for agricultural products. In terms of biogeochemical flows, China had the largest amount of unsustainable nitrogen applications, substantially higher than India who ranked the second, while India used the largest amount of unsustainable phosphorus, closely followed by China. It should be noted that national planetary boundaries are determined by national resource endowments, e.g., water availability and arable land, while national planetary transgressions are affected by the population size as larger populations are associated with higher demands on natural resources and therefore more likely to transgress the national planetary boundaries.

### Sustainable food production for reducing pressure on planetary and national boundaries

Gerten et al. (2020)^20^ have pointed out that if national boundaries were strictly respected, the current food systems could provide a balanced diet for 3.4 billion people only worldwide. However, food systems transformation toward more sustainable production and consumption patterns could support more than 10 billion people within the planetary boundaries. Sustainable agricultural practices could be promoted in the major countries where food production is causing substantial transgression of national boundaries, such as irrigation water saving in India, China, Pakistan, the US, and Egypt; agricultural land conservation in the US, China, Brazil, Australia, and Kazakhstan; and sustainable fertilization in China, India, Brazil, and the US. Hu et al. (2020)^28^ concluded that 47 to 99 percent reduction in phosphorus and nitrogen emissions, greenhouse gas emissions, and water and cropland use is needed for China’s food production to be within national and provincial environmental boundaries by 2030.

Some predominant agricultural water-saving technologies include drip and intermittent irrigation, chemical-based sprays, and so forth.^29^ Plastic mulching combined with subsurface drip irrigation is shown to offer potentials to reduce water consumption by about 7 percent in arid regions.^30^ In order to meet increasing food demands with limited land resources, conservation agriculture is a widely promoted concept that aims to prevent arable land losses while regenerating degraded lands. It promotes maintenance of a permanent soil cover, minimum soil disturbance, and diversification of plant species, which also contributes to increased water and nutrient use efficiency as well as productivity improvement.^31^ Measures to reduce nutrition runoff include water and soil conservation, chemical fertilizer reduction, and so forth. Gerten et al. (2020)^20^ estimated that a combination of environment-sensitive production technology improvement could reduce global agricultural areas by 16 percent, irrigation water use by 7 percent, and nitrogen fertilization by 38 percent. Besides the aforementioned technical measures, financial measures could also be promoted, such as increasing water tariffs to reflect local water scarcity, removing subsidies for fertilizer, etc.^32^

### Sustainable food consumption for reducing life cycle pressure on national boundaries in other countries

The environmental impacts of food systems could be minimized from both producer and consumer’s sustainable practices. From the consumer’s perspective, environmentally responsible food choices should be promoted. For instance, the increasing consumption of avocado by bourgeoning urbanites is imposing increasing water pressure along the life cycle supply chain.^33^ Environmental information disclosure and raising awareness such as via environmental labeling could be a useful way to change people’s consumption behaviors. Information such as transgression of planetary boundary for water along the supply chain can be added to green labels for traded products to inform consumer behaviors.

On other note, food demand management is of critical importance of reaping the double benefits of eliminating hunger while minimizing environmental disturbances.^34^ One effective way to address food security while also reducing planetary boundary transgression is to diminish over-consumption and food waste. According to Food and Agriculture Organization (FAO) estimation, the global volume of food waste was estimated to be 1.3 billion tons, which accounted for 3.3 billion tons of greenhouse gas emissions, 250 km^3^ of water lost, and 1.4 billion hectares of land wasted.^35^ Last but not the least, changing dietary patterns may also offer potentials to reduce planetary boundary transgression without compromising food security goals. A systematic review estimated that shifts in consumption could reduce diet-related land use demand by up to 50 percent.^36^ Furthermore, food consumption management or transition in dietary patterns in developed countries, such as Germany and Japan, could contribute to alleviation of planetary boundary transgression in their trading partners, for instance, water crisis in Pakistan (Figure 3).

### Conclusion

We quantify unsustainable environmental impacts of national food systems from both production and consumption perspectives of 189 countries/regions around the world with a global multi-regional input-output (MRIO) analysis. Unsustainable environmental impacts are quantified based on the exceedance of national planetary boundaries, and the cross-national transferred environmental impacts from consumers to producers are also evaluated. Unsustainable amounts of nitrogen were used in China and the US to support food consumption in many other countries, including Japan, South Korea, and others, in line with findings from Shi et al. (2016).^37^ With growing food demands, transgression of national boundaries caused by the global food system has aggravated over time. However, meanwhile, global trade has avoided more unsustainable water use in 2015 (270 km^3^) than in 1995 (218 km^3^), but less unsustainable nitrogen (18 million tons in 2015 and 29 million tons in 1995), phosphorus (7 million tons in 2015 and 13 million tons in 1995), and land uses (5.4 billion km^2^ in 2015 and 14.3 billion km^2^ in 1995). Literature review in the virtual water field has also concluded that food trade has generally led to global water savings.^38^ Alleviation of planetary boundary transgression has been achieved primarily in the US, China, Saudi Arabia, etc., while aggravation was incurred in Pakistan, Australia, Argentina, and so forth.

Trade patterns can be optimized to internalize its environmental externalities. Although optimized trade needs to consider a large variety of factors, including predominantly capital, labor, and technology among others, environmental impacts, especially those that have already transgressed national boundaries where products are produced, should also be incorporated. National boundary transgression provides another important parameter measuring unsustainable environmental impacts induced by trade. Our results have shown that although environmental impacts of food productions are still well within national boundaries for some, i.e., Canada and North European countries, national boundaries of their trading partners, e.g., Brazil, have been seriously transgressed (Figure 2). On another note, this study has also shown that trade has contributed to alleviation of national boundary transgression on a global scale but with aggregation in different countries and therefore induced important considerations on inequality issues.

### Limitations of the study

However, it should be noted that there are several limitations and uncertainties in the methodology and therefore results achieved in this study, which could be further studied in future academic endeavors. First, MRIO uses sector average environmental impacts intensity data, which may generate uncertainties in estimating country-specific links and transfers. Secondly, downscaling from global to national values can be conducted through different ways, which all entail methodological uncertainties. Due to data availability, we distribute planetary boundary to national level according to farmland area. However, such approach can create perverse incentives for countries who have deforested a lot of land today than countries that retained their intact forests because the former gets higher shares of the planetary boundary. Some of these issues could be avoided in future studies, for example, by using regional planetary boundaries.^20^^,^^39^ Thirdly, it should be noted that there is a “zone of uncertainty” within the planetary boundaries identified, beyond which the risk increases before the boundary is fully transgressed.^40^ Assessments of transgressions of the boundaries themselves as well as of the uncertainty zones in future studies could provide a range of risk-informed decision-making recommendations.

## STAR★Methods

### Key resources table

**Table:** 

REAGENT or RESOURCE	SOURCE	IDENTIFIER
**Deposited data**		

National boundaries for food system	This paper	Figure S1

**Software and algorithms**		

MATLAB	MathWorks	https://www.mathworks.com/products/matlab.html

### Resource availability

#### Lead contact

Further information and request for resources should be directed to and would be fulfilled by the Lead Contact, Li Chai (chaili@cau.edu.cn).

#### Materials availability

All newly created databases of this study can be found in supplemental information.

### Method details

#### National environmental boundaries

Planetary boundaries delineate safe operational spaces for maintaining the stability of earth systems. The national boundaries for food systems are the environmental limits that a country can use for sustainable food production. This study considers four national boundaries for food systems, i.e., National Boundary for Water (NBW), National Boundary for Land (NBL), National Boundary for Nitrogen (NBN), and National Boundary for Phosphorous (NBP).

Similar with Hu et al. (2020),^30^ we downscale the global planetary boundaries for food systems assessed by Springmann et al. (2018)^11^ to the country level using a multi-scale approach by incorporating the regional resource endowments.^41^ The national boundaries can be quantified using the product of the planetary boundaries and a corresponding proxy ratio, i.e., the regional proxy divided by the global total. For the calculations of NBW, NBN, and NBP, the average national total renewable water availability over the last 30 years is used as the proxy. For NBL, the national farmland area is used. Water availability and farmland area data can be acquired from the Food and Agriculture Organization (FAO). Source of the data of this study can be found in Table S1, and the four downscaled boundaries are shown in the Figure S1.

It should be noted that while some previous studies, such as Hu et al. (2020),^30^ determined the national boundaries for nitrogen and phosphorous using the discharge amounts of nitrogen and phosphorous to water bodies, we used the application amounts of nitrogen and phosphorous, same as Springmann et al. (2018),^11^ due to the methodological difficulties and uncertainties in determining the leaching ratios of nitrogen and phosphorous throughout the globe.

#### Multi-regional input-output model

A Multi-Regional Input-Output (MRIO) model is used to assess the global transfers of unsustainable environmental impacts (i.e., unsustainable water use, unsustainable land use, unsustainable nitrogen application and unsustainable phosphorous application) as virtual flows embodied in global food trade. Unsustainable environmental impacts are defined as those exceeded, or transgressed, national boundaries (i.e., NBW, NBL, NBN and NBP). We adopt a highly disaggregated MRIO database (189 countries/regions and 163 sectors) complied by Cabernard and Pfister (2020)^42^ from 1995 to 2015. There are 24 food-related sectors in the MRIO tables studied and shown in the Table S2. According to the Input-Output Framework, the environmental footprints for traded food products can be calculated as the following:

(Equation 1) $$EF={D\left(I-A\right)}^{-1}$$

where, EF is the life-cycle environmental footprints (i.e., water, land, nitrogen, or phosphorus) coefficient matrix comprised of ${footprint}_{i}^{r}$; A is the direct consumption coefficient matrix that can be obtained from the MRIO table; I is an identify matrix; D is the matrix of direct use coefficient of each environmental indicator, which is obtained from the following sources. The Cabernard and Pfister (2020)^42^ database included the data of blue water use and land use but no data for nitrogen and phosphorous use. We adopted the data of nitrogen and phosphorous use of 49 regions from EXIOBASE^43^ and then disaggregated them into 189 countries/regions by the same approach of Cabernard and Pfister (2020).^42^

The unsustainable environmental impacts, i.e. environmental footprints that have transgressed national boundaries (${footprint}_{tran}$), can be quantified by subtracting the corresponding national boundaries from the respective environmental footprint that is used by food production in a certain region or required to meet food demands of a certain region (${footprint}_{tran}$ equals zero if the environmental footprint is smaller than boundary).

The global virtual flows of unsustainable environmental impacts can be assessed by the below Equation 2.

(Equation 2) $${flow}^{r,s}=\sum_{i}{{footprint}_{tran}}_{i}^{r}\times y_{i}^{r,s}$$

where, ${flow}^{r,s}$ represents the transgressed virtual (water, land, N, or P) flows from region r to s; $y_{i}^{r,s}$ indicates the final consumption of sector i that is produced in region r and finally consumed by region s; i is the food sector as listed in the Table S2. The exports and imports of transgressed virtual resource can be estimated based on the transgressed flow results (${flow}^{r,s}$).

In the scenario of “no trade”, we assume that all consumed foods were produced domestically, which can be calculated by $\sum_{i}{{footprint}_{trans}}_{i}^{s}\times y_{i}^{s}$. If a certain type of food is not produced in a country (e.g., some countries do not grow paddy rice), the global average value of environmental footprint of this food is adopted to perform the assessment of this country in the scenario of “no trade”. The methodological framework of this study is shown in the Figure S2.

## Data Availability

The Multi-Regional Input-Output database adopted in this study can be found at https://doi.org/10.5281/zenodo.3993659. The codes and more data of this study can be found at https://zenodo.org/record/8143907. All the datasets are publicly accessible, and any additional information required to reanalyze the data reported in this paper is available from the lead contact upon request.
