# Supplementary material for: Global food trade alleviates transgressions of planetary boundaries at the national scale
Source: iScience. 2023 Aug 30;26(10):107794. doi: 10.1016/j.isci.2023.107794 (PMC10504541; doi:10.1016/j.isci.2023.107794)
Supplement: Document S1. Figures S1 and S2 and Tables S1–S3 [file mmc1.pdf]

iScience, Volume *26*

Supplemental information

Global food trade alleviates transgressions of planetary boundaries at the national scale

Xiawei Liao, Ao Liu, and Li Chai

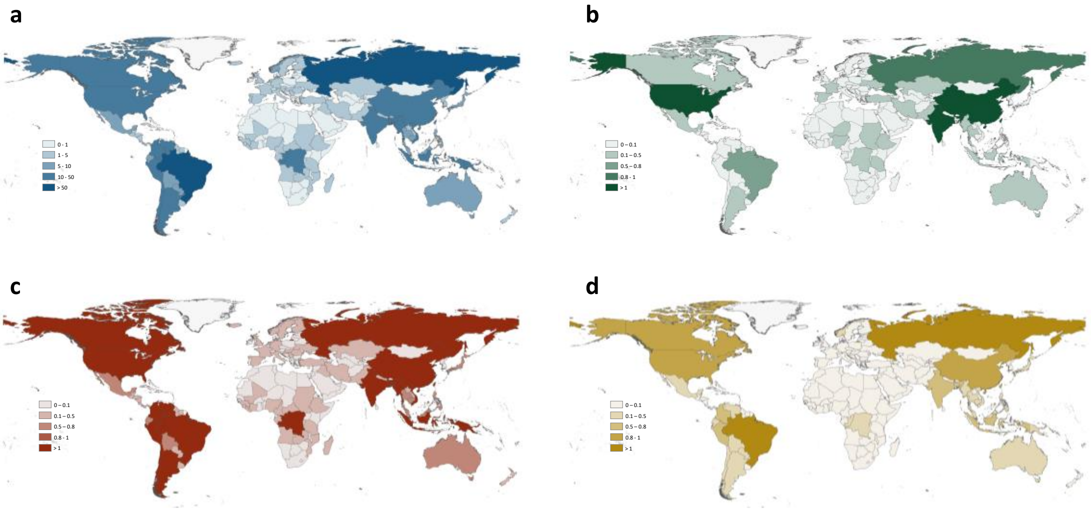

Supplementary Figure 1. National boundaries for food system. a) Freshwater (km^3); **b)** Land (Mkm^2); **c)** Nitrogen (Tg) and **d)** Phosphorus (Tg), related to STAR Methods.

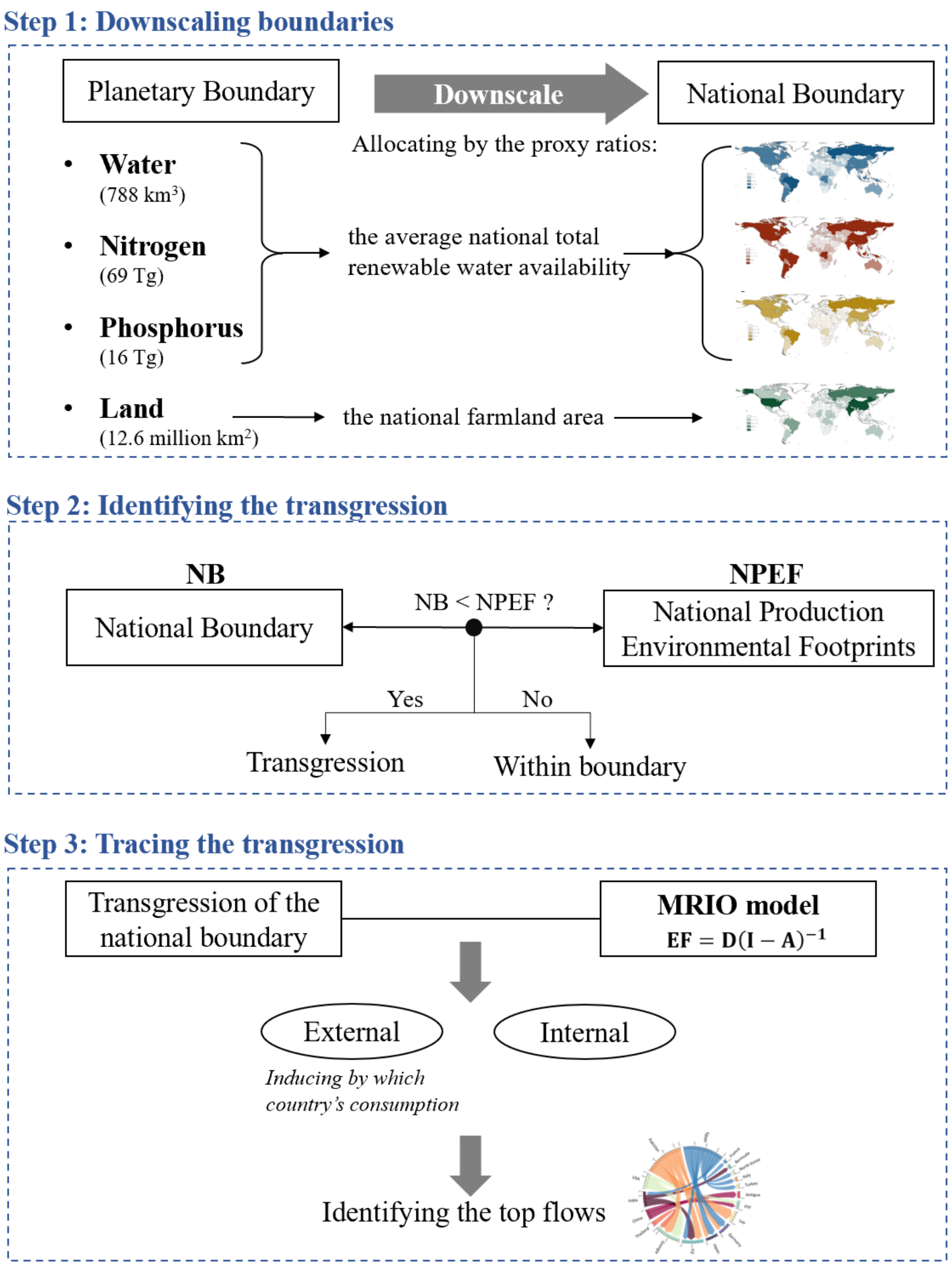

Supplementary Figure 2. Methodological framework of this study, related to STAR Methods.

Supplementary Table 1. Sources of the data used in the study, related to STAR Methods.

Data	Source
MRIO table (189 regions×163 sectors)	Cabernard and Pfister, 2020
Bluewater use (sector-wise data at the country level)	Cabernard and Pfister, 2020
Land use (sector-wise data at the country level)	Cabernard and Pfister, 2020
N application (sector-wise data at the country level)	Stadler et al., 2021
P application (sector-wise data at the country level)	Stadler et al., 2021
Planetary boundary for bluewater footprint	Hu et al., 2020
Planetary boundary for nitrogen application	Springmann et al., 2018
Planetary boundary for phosphorus application	Springmann et al., 2018
Planetary boundary for cropland use	Springmann et al., 2018
Water availability (country level)	FAO database
Farmland area (country level)	FAO database

Supplementary Table 2. Food-related sectors in the Multi-Regional Input-Output tables of this study, related to STAR Methods.

1	Cultivation of paddy rice
2	Cultivation of wheat
3	Cultivation of other cereal grains
4	Cultivation of vegetables, fruit, nuts
5	Cultivation of oil seeds
6	Cultivation of sugar cane, sugar beet
7	Cultivation of other crops
8	Cattle farming
9	Pigs farming
10	Poultry farming
11	Other meat animals
12	Other animal products
13	Raw milk
14	Processing of meat cattle
15	Processing of meat pigs
16	Processing of meat poultry
17	Production of other meat products
18	Processing vegetable oils and fats
19	Processing of dairy products
20	Processed rice
21	Sugar refining
22	Processing of other food products
23	Manufacture of beverages
24	Manufacture of fish products

Supplementary Table 3. Global transgression of national boundaries in different scenarios in 2015, related to Figure 4.

Item		Global transgression in the current scenario	Global transgression in "no trade" scenario
Water	km^3	713	983
N	million tons	31	49
P	million tons	27	34
Land	million km^2	22850	28281
